# Generation of a lethal mouse model expressing human ACE2 and TMPRSS2 for SARS-CoV-2 infection and pathogenesis

**DOI:** 10.1038/s12276-024-01197-z

**Published:** 2024-05-31

**Authors:** Gi Uk Jeong, Insu Hwang, Wooseong Lee, Ji Hyun Choi, Gun Young Yoon, Hae Soo Kim, Jeong-Sun Yang, Kyung-Chang Kim, Joo-Yeon Lee, Seong-Jun Kim, Young-Chan Kwon, Kyun-Do Kim

**Affiliations:** 1 Center for Infectious Disease Vaccine and Diagnosis Innovation (CEVI), Korea Research Institute of Chemical Technology, Daejeon, Republic of Korea; 2 Center for Emerging Virus Research, National Institute of Health, Korea Disease Control and Prevention Agency, Cheongju, Republic of Korea; 3 Medical Chemistry and Pharmacology, University of Science and Technology (UST), Daejeon, Republic of Korea; 4 Present Address: Division of Infectious Diseases, Department of Pediatrics, Boston Children’s Hospital, Boston, MA USA; 5 Present Address: Department of Pediatrics, Harvard Medical School, Boston, MA USA; 6 Present Address: Division of Vaccine Development Coordination, Center for Vaccine Research, National Institute of Infectious Diseases, National Institute of Health, Korea Disease Control and Prevention Agency, Cheongju, Republic of Korea

**Keywords:** Viral infection, Transgenic organisms

## Abstract

Mouse models expressing human ACE2 for coronavirus disease 2019 have been frequently used to understand its pathogenesis and develop therapeutic strategies against SARS-CoV-2. Given that human TMPRSS2 supports viral entry, replication, and pathogenesis, we established a double-transgenic mouse model expressing both human ACE2 and TMPRSS2 for SARS-CoV-2 infection. Co-overexpression of both genes increased viral infectivity in vitro and in vivo. Double-transgenic mice showed significant body weight loss, clinical disease symptoms, acute lung injury, lung inflammation, and lethality in response to viral infection, indicating that they were highly susceptible to SARS-CoV-2. Pretreatment with the TMPRSS2 inhibitor, nafamostat, effectively reduced virus-induced weight loss, viral replication, and mortality in the double-transgenic mice. Moreover, the susceptibility and differential pathogenesis of SARS-CoV-2 variants were demonstrated in this animal model. Together, our results demonstrate that double-transgenic mice could provide a highly susceptible mouse model for viral infection to understand SARS-CoV-2 pathogenesis and evaluate antiviral therapeutics against coronavirus disease 2019.

## Unveiling susceptibility of SARS-CoV-2 variants in double-transgenic mice

This research aimed to create a type of mouse that is very prone to SARS-CoV-2 infection, the virus that causes COVID-19. Scientists from two Korean research institutes found that mice with both human angiotensin-converting enzyme 2 (hACE2, a protein that the virus uses to enter cells) and human transmembrane serine protease 2 (hTMPRSS2, an enzyme that helps the virus to infect cells) were very prone to the virus. They created mice with both these human genes, infected them with different amounts of SARS-CoV-2, and observed significant weight loss, disease symptoms, and death, similar to what happens in COVID-19 patients. The scientists concluded that these mice could help us understand how the virus infects and causes disease, and test potential treatments against COVID-19. The study could also help in developing future drugs and vaccines.

This summary was initially drafted using artificial intelligence, then revised and fact-checked by the author.

## Introduction

Since the coronavirus disease 2019 (COVID-19) outbreak, the development of therapeutic agents and vaccines is still needed to alleviate the current pandemic. Several animal models of severe acute respiratory syndrome coronavirus 2 (SARS-CoV-2) infection have been used to study viral pathogenesis and transmission, including nonhuman primates, ferrets, and Syrian hamsters^1,2^. However, these models do not reflect the severity or mortality of COVID-19. The K18-hACE2 transgenic mouse, in which human angiotensin-converting enzyme 2 (hACE2) is expressed, is most commonly used for SARS-CoV-2 infection because murine ACE2 does not effectively bind to the viral spike protein^3^. This transgenic mouse provides a model of clinical disease and lethal infection, allowing the effective evaluation of therapies and vaccines^4–6^. These studies suggest that a mouse model that is highly susceptible to viral infection is helpful for the development of therapeutic agents and vaccines.

The SARS-CoV-2 spike protein consists of S1 and S2 proteins, which function in receptor recognition and mediate membrane fusion^4,5^. Cleavage of S1/S2 by proteases promotes binding of the spike protein to ACE2 and causes cleavage of the S2′ site^6^. As transmembrane serine protease 2 (TMPRSS2), a type II transmembrane serine protease, supports viral entry via proteolytic activation of the SARS-CoV-2 spike protein^7–9^, the TMPRSS2-expressing Vero E6 cell line was reported to be highly susceptible to SARS-CoV-2 infection^10^. TMPRSS2 is also involved in viral replication, pathogenesis, and host immune responses^11^.

In this study, we established a SARS-CoV-2 infection mouse model expressing both hACE2 and hTMPRSS2, which was expected to be highly susceptible to viral infection. The double transgenes, hACE2 and hTMPRSS2, were simultaneously microinjected to generate double-transgenic (double-Tg) mice. We then evaluated the susceptibility, viral replication, pathological symptoms, and cytokine and chemokine levels in mouse lungs following SARS-CoV-2 and variant infection. In addition, we determined whether double-Tg mice could be used to develop potential therapeutic agents.

## Materials and methods

### Biosafety

All procedures were performed at a biosafety level 3 (BSL-3) or an animal BSL-3 facility for SARS-CoV-2-related experiments by personnel equipped with powered air-purifying respirators. This study was approved by the Institutional Animal Care and Use Committee of the Korea Research Institute of Chemical Technology (protocol IDs: 8A-M6, IACUC ID 2021-8A-02-01, and 2021-8A-03-03).

### Cells

A549 cells (CCL-185) were purchased from the American Type Culture Collection (ATCC; Manassas, VA, USA). The cell line was maintained at 37 °C in Dulbecco’s Modified Eagle medium (Cytiva, Marlborough, MA, USA) containing 10% fetal bovine serum (Gibco, Waltham, MA, USA) and 1% penicillin/streptomycin (Gibco).

### Mice

C57BL/6 mice were purchased from Orient Bio, Inc. (Gyeonggi-do, Republic of Korea). A group of 8–12-week-old male and female double-Tg mice were intranasally administered SARS-CoV-2 inocula (2 × 10^3^ plaque-forming units [PFU]) under anesthesia using isoflurane in a BSL-3 animal facility. The mock group was injected with the same volume of phosphate-buffered saline (PBS) in all experiments. Mice were monitored and weighed daily. Clinical disease symptoms were scored from 0–4 as follows: 0, no symptoms; 1, ruffled fur; 2, reduced mobility; 3, hunched posture; and 4, moribund or death. In this study, 30% weight loss was considered the humane euthanasia criterion via CO_2_ asphyxiation, except during the first mouse lethal dose 50 (MLD_50_) assessment. Organ tissues were collected at the indicated days post infection (dpi) after the animals were anesthetized with isoflurane, followed by transcardial perfusion with cold PBS. The tissues were weighed and homogenized in preloaded steel bead tubes containing cold PBS using a tacoPrep Bead Beater (GeneReach Biotechnology Corp., Taichung City, Taiwan).

### Lentivirus-based pseudovirus production

A plasmid kit (NR-52948) producing a lentivirus-based pseudovirus (PV) with the SARS-CoV-2 spike protein (GenBank: NC_045512) and luciferase gene was obtained from BEI Resources (NIAID, NIH). PV-containing supernatants were collected from plasmid-transfected HEK293T cells and titrated as previously reported^7,8^.

### Viruses

SARS-CoV-2 (GISAID: EPI_ISL_407193) and its variants (Beta, NCCP 43382; Omicron, NCCP 43408) were obtained from the Korea Centers for Disease Control and Prevention (KCDC) and propagated in Vero cells (CCL-81; ATCC). Culture supernatants containing the viruses were stored at −80 °C. Viral titers were measured using a plaque assay as described previously^9^.

### TMPRSS2 inhibitor treatment

The 8–12-week-old female double-Tg mice were intranasally administered 3 mg/kg nafamostat mesylate (N0289; Sigma-Aldrich, St. Louis, MO, USA) 2 h before SARS-CoV-2 infection (2 × 10^3^ PFU) under anesthesia using isoflurane in a BSL-3 animal facility. The vehicle group was injected with an equal volume of 0.9% normal saline (Daihan Pharm. Co., Ltd., Gyeonggi-do, Korea).

### In vitro and in vivo transfection

Cells (2 × 10^5^ cells per well) were plated into 6-well plates and transfected with 2 μg pCMV3-hACE2-FLAG (NM_021804.1, HG10108-CF; Sino Biological, Beijing, China) and/or 0.3 μg pCMV3-hTMPRSS2-HA (NM_005656.3, HG13070-CY; Sino Biological) using a TransIT-LT1 Transfection Reagent (Mirus Bio, Madison, WI, USA) according to the manufacturer’s recommendations.

For in vivo transfection, 6-week-old male and female C57BL/6 mice were intranasally transfected with 40 μg pCMV3 vector or pCMV3-hACE2-FLAG, or co-transfected with 30 μg pCMV3-hACE2-FLAG and 10 μg pCMV3-hTMPRSS2-HA using the in vivo-jetPEI reagent (Polyplus, Illkirch, France) in two doses 1 h apart (each 50 μl) per the manufacturer’s instructions (N/P ratio = 8). Mice were infected with 5 × 10^5^ PFU SARS-CoV-2 for subsequent experiments.

### Plaque assay

The mouse lung homogenates were serially diluted in Eagle’s minimum essential medium supplemented with 2% fetal bovine serum for the plaque assay. The dilutions were transferred onto Vero E6 cell monolayers in a 24-well plate (~1 × 10^5^ cells/well). After incubation at 37 °C for 1 h, the infected cells were washed with PBS and overlaid with 1.8% carboxymethyl cellulose in the minimal essential medium. The samples were incubated for 4 days, followed by fixation and staining with 0.05% crystal violet containing 1% formaldehyde. The plaques were counted and measured using ImmunoSpot version 5.0 software and an analyzer (Cellular Technology Ltd., Shaker Heights, OH, USA).

### RNA extraction and RT-qPCR

Total cellular RNA was extracted using the RNeasy Mini Kit (QIAGEN, Hilden, Germany). RNA was extracted from tissue homogenates using the Maxwell RSC simplyRNA tissue kit (Promega, Madison, WI, USA) following the manufacturer’s protocol. Quantitative RT-PCR (QuantStudio 3; Applied Biosystems, Foster City, CA, USA) was performed using a one-step Prime Script III RT-qPCR mix (Takara, Kyoto, Japan). Viral RNA of the nucleocapsid protein (NP) was detected using a 2019-nCoV RUO kit (10006713; Integrated DNA Technologies, Coralville, IA, USA). The primers and probe specific for hACE2 were as follows: forward primer, 5′-GCCACTGCTCAACTACTTTG-3′; reverse primer, 5′-GCTTATCCTCACTTTGATGCTTTG-3′; and probe, 5′-ACTCCAGTCGGTACTCCATCCCA-3′. Those for hTMPRSS2 were as follows: forward primer, 5′-TGTACTCATCTCAGAGGAAGTCC-3′; reverse primer, 5′-CTGGTGGATCCGCTGTC-3′; and probe, 5′-ACCCTGTGTGCCAAGACGACT-3′. Absolute quantification of hACE2 and hTMPRSS2 mRNA copies was performed by constructing each standard curve using serial dilutions of pCMV3-hACE2-FLAG and pCMV3-hTMPRSS2-HA.

### Western blotting

Proteins in the lysate were separated on a denaturing polyacrylamide gel and transferred onto a polyvinylidene fluoride (PVDF) membrane (Merck Millipore, Burlington, MA, USA). The membrane was incubated with 5% skim milk (BD Biosciences, Franklin Lakes, NJ, USA) in Tris-buffered saline with 0.1% Tween 20 (TBST) buffer and the primary antibodies; namely, anti-hACE2 (Abcam, Cambridge, UK), anti-FLAG M2 (Sigma-Aldrich), anti-HA-Tag (6E2; Cell Signaling Technology), and anti-GAPDH (14C10; Cell Signaling Technology). Horseradish peroxidase (HRP)-conjugated secondary antibodies (Bio-Rad, Hercules, CA, USA) and enhanced chemiluminescence (ECL) reagents (Thermo Fisher Scientific, Waltham, MA, USA) were used for protein detection.

### Multiplex immune analysis

The lungs of SARS-CoV-2-infected mice were dissected at 0, 3, and 6 dpi and then homogenized in bead tubes, followed by incubation in Triton X-100 (1% final concentration) for 16 h at 4 °C to inactivate the virus. Aliquots were analyzed using the MILLIPLEX human cytokine/chemokine magnetic bead panel (HCYTOMAG-60K; Merck Millipore) with Luminex 200 multiplexing instruments (40-012; Merck Millipore) to assess cytokine/chemokine expression.

### Histology and immunohistochemistry

Mice were anesthetized and transcardially perfused with cold PBS. The lungs were harvested and inflated with 10% neutral-buffered formalin for 16 h at 24 °C. Samples were processed routinely and embedded in paraffin wax (Leica, Wetzlar, Germany). The tissues were cut into 5-μm sections and stained with hematoxylin and eosin (BBC Biochemical, Mount Vernon, WA, USA) using an autostainer (Leica). Serial sections were immunostained with anti-SARS-CoV-2 NP rabbit monoclonal antibody (40143-R001; Sino Biological). Images were captured using an Olympus BX51 microscope (Olympus, Tokyo, Japan) and Nuance 3.02 software (PerkinElmer, Waltham, MA, USA).

### Statistical analysis

All experiments were performed at least three times. All data were analyzed using the GraphPad Prism 8.0 software (GraphPad Software, San Diego, CA, USA). Statistical significance was set at *p* < 0.05. The specific analytical methods used are described in the figure legends.

## Results

### Both in vitro and in vivo transfection of hACE2 and hTMPRSS2 increased viral infection

**Fig. 1:**
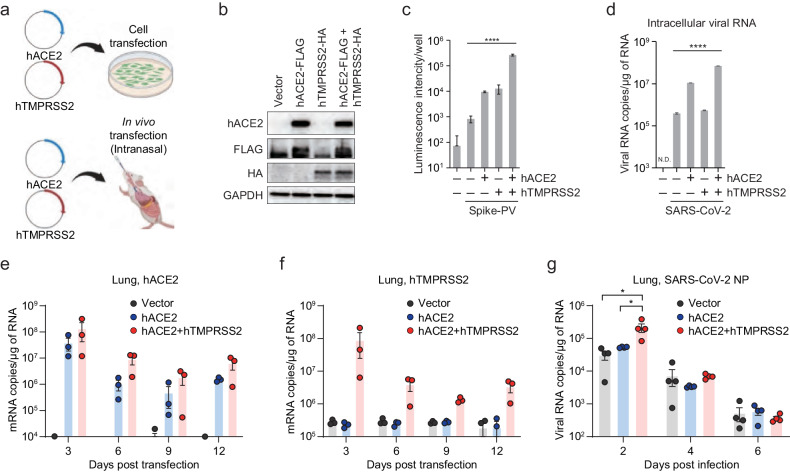
Augmented viral burden of SARS-CoV-2 by in vitro and in vivo co-transfection of hACE2 and hTMPRSS2. **a** An experimental graphic created with BioRender.com. A549 cells or C57BL/6 mice were transfected with plasmid DNA as indicated to overexpress hACE2 and hTMPRSS2 in vitro and in vivo, respectively. **b** Plasmids expressing hACE2-FLAG (2 μg) or hTMPRSS2-HA (0.3 μg) were transfected into A549 cells on a 6-well plate. Overexpressed proteins at 24 h post transfection were analyzed using western blotting. GAPDH served as a loading control. **c** At 24 h post-transfection of plasmids, the cells were incubated with SARS-CoV-2-PV (1.3 × 10^5^ TCID50/ml). After 24 h, the infectivity of the pseudovirus (PV) was assessed by measuring relative luminescence intensity. **d** At 2 days post-transfection, the cells were infected with SARS-CoV-2, followed by a further 2 days of incubation. The intracellular RNA was extracted and used to detect viral RNA of the SARS-CoV-2 nucleocapsid protein (NP) via RT-qPCR. **e**–**g** In vivo-transfected mice were intranasally infected with SARS-CoV-2 (5 × 10^5^ PFU). Expression levels of hACE2 (**e**), hTMPRSS2 (**f**), and viral load (**g**) in the lungs were measured at the indicated days post-infection using RT-qPCR. Symbols represent the means ± SEM. Statistically significant differences between the groups were determined using one-way ANOVA (**c**, **d**) or unpaired two-tailed *t*-tests (**g**).

Given that hACE2 and hTMPRSS2 are involved in the entry step of SARS-CoV-2 infection, we examined whether their co-expression could augment viral infection via in vitro and in vivo transfection (Fig. 1a). First, we transiently co-transfected hACE2 and hTMPRSS2 into human alveolar A549 cells that express negligible levels of these genes. After 24 h, the expression levels of hACE2 and hTMPRSS2 were determined using western blotting (Fig. 1b). To verify their role in viral entry, we used lentivirus-based PVs expressing luciferase and pseudotyped them with the S protein of SARS-CoV-2 as previously described^7,8^. The results revealed that the PV entry was most efficient in hACE2 and hTMPRSS2 co-transfected cells (Fig. 1c). Next, the transfected cells were infected with SARS-CoV-2 at a multiplicity of infection of 1. Viral RNA levels in cell lysates and culture media showed the strongest increase after hACE2 and hTMPRSS2 co-expression, as shown via RT-qPCR analysis (Fig. 1d). Second, we transfected both genes in vivo into C57BL/6 mice via the intranasal route. The expression of hACE2 and hTMPRSS2 mRNA in the lungs decreased over time but remained at low levels until 12 dpi, as shown in Fig. 1e, f. When the transfected mice were intranasally infected with 1 × 10^4^ PFU SARS-CoV-2, the co-expressing mice were more susceptible to viral infection at 2 dpi than the single-expressing mice (Fig. 1g). However, viral RNA levels rapidly decreased with time as the expression of hACE2 and hTMPRSS2 declined due to transient transfection. These results suggest the need to generate double-Tg mice to further evaluate their susceptibility to SARS-CoV-2 infection.

### SARS-CoV-2 infection in hACE2 and hTMPRSS2 double-transgenic mice

**Fig. 2:**
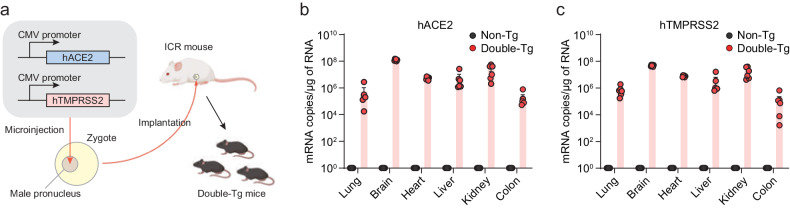
hACE2 and hTMPRSS2 expression in the double-transgenic (double-Tg) mice. **a** Experimental graphic for generating the double-Tg mice. Expression levels of hACE2 (**b**) and hTMPRSS2 (**c**) mRNA in the lungs, brain, heart, liver, kidney, and colon of the double-Tg or wild-type C57BL/6 mice (non-Tg) were assessed via RT-qPCR. Symbols represent the means ± SEM.

**Fig. 3:**
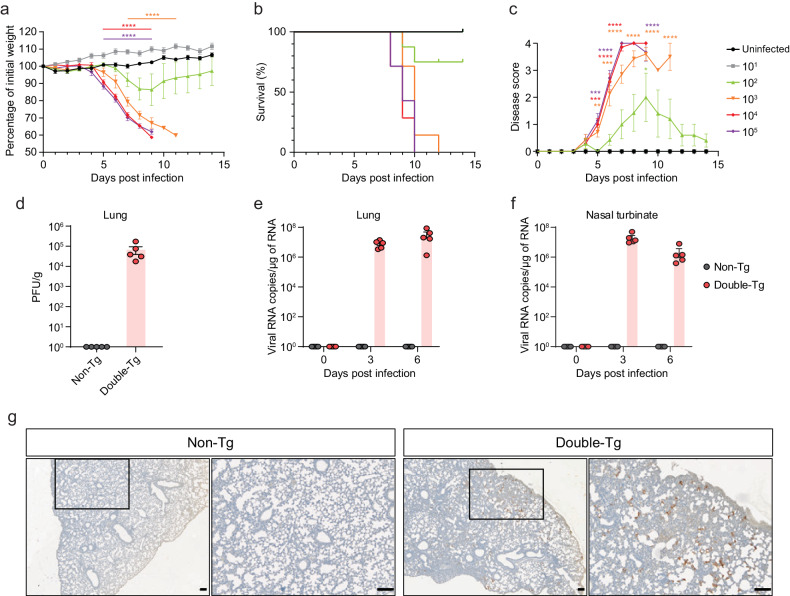
SARS-CoV-2 infection in the double-transgenic (double-Tg) mice. Percentage of initial weight (**a**), survival rate (**b**), and clinical disease symptoms (**c**) of the double-Tg mice infected with 1 × 10^1^–10^5^ PFU SARS-CoV-2. Clinical disease symptoms were scored as described in the “Materials and methods”. Infectious viral titers in the lungs (**d**) and viral burdens in the lungs (**e**) and nasal turbinate (**f**) of the double-Tg mice infected with 2 × 10^3^ PFU SARS-CoV-2 (6 days post-infection; dpi) were detected by using a plaque assay and RT-qPCR, respectively. **g** Representative images of SARS-CoV-2 nucleocapsid protein (NP) immunostaining in the lungs of C57BL/6 (non-Tg) and double-Tg mice. Images show low (left; bars, 100 μm) and high magnification (right; bars, 100 μm). Symbols represent means ± SEM. Statistically significant differences between the groups were determined using multiple two-tailed *t*-tests (**a**, **c**).

To generate double-Tg mice bearing both hACE2 and hTMPRSS2 under the control of the CMV promoter, these transgenes were simultaneously microinjected into the male pronucleus of fertilized eggs from C57BL/6 mice, followed by transgenic zygote transfer and delivery (Fig. 2a). The expression levels of hACE2 and hTMPRSS2 were detected with similar expression patterns in several tissues, including the lungs, brain, heart, liver, kidney, and colon (Fig. 2b, c). Wild-type C57BL/6 (non-Tg) mice served as negative controls. Next, double-Tg mice were intranasally infected with different viral doses of SARS-CoV-2 to determine the MLD_50_. Body weights, survival rates, and clinical symptoms were monitored and shown to increase in a dose-dependent manner (Fig. 3a–c). All mice infected with over 1 × 10^3^ PFU inocula began to lose weight, became lethargic by 5 dpi, and eventually succumbed to the disease by 9–12 dpi, whereas all mice infected with 1 × 10 PFU survived. Mice infected with 1 × 10^2^ PFU showed variable mortality (3 out of 4 females and 1 out of 3 male mice survived). The MLD_50_ to SARS-CoV-2 in double-Tg mice was calculated to be 2 × 10^2^ PFU using the Reed and Muench method^12,13^. This MLD_50_ measurement was comparable with that of other transgenic mice, suggesting that double-Tg mice are highly susceptible to SARS-CoV-2^14–16^. Thus, all mice were infected with 10 MLD_50_ (2 × 10^3^ PFU) in subsequent experiments. Infectious virus particles in the lungs were assessed at 6 dpi using a plaque assay (Fig. 3d). The tissue distribution of viral RNA in the lungs, nasal turbinate, brain, heart, liver, kidney, spleen, intestine, and colon at 3 and 6 dpi was determined using RT-qPCR. Viral titers in the lungs increased with time, whereas those in the nasal turbinate declined (Fig. 3e, f). The predominant tissue distributions were in the brain and lungs as target organs (Supplementary Fig. 1). We also detected the nucleocapsid protein in lung sections derived from SARS-CoV-2-infected double-Tg mice using immunohistochemistry (Fig. 3g). These results demonstrate that the double-Tg mice are highly susceptible to SARS-CoV-2 infection and that the lungs and brain are the main targets of viral infection.

### Inflammatory and pathological changes in the lungs of SARS-CoV-2-infected double-transgenic mice

**Fig. 4:**
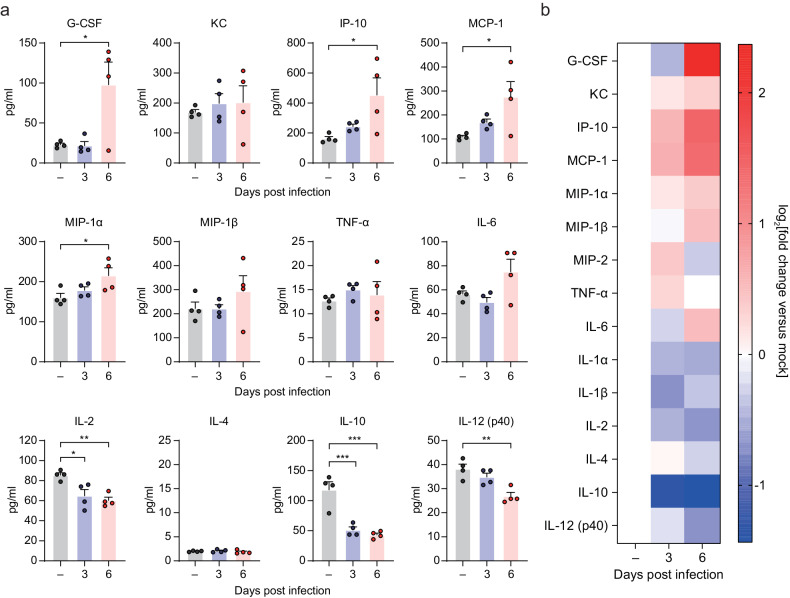
Cytokine and chemokine analysis following SARS-CoV-2 infection in the lungs of the double-transgenic (double-Tg) mice. **a** Cytokine and chemokine protein levels in the lungs of the double-Tg mice were measured using multiplex immune analysis at 0 (mock), 3, and 6 days post-infection. Symbols represent means ± SEM. Statistically significant differences between the groups were determined using unpaired two-tailed *t*-tests. **b** Heat map of cytokine and chemokine analysis results as measured with multiplex immune analysis. The fold change was calculated by comparing expression levels of the double-Tg mice with those of the mock group and log_2_[fold change] shown in the heat map.

**Fig. 5:**
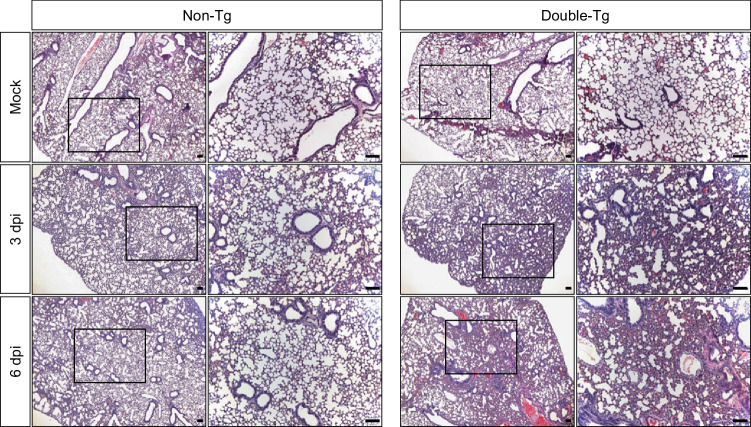
Histopathological changes in non-transgenic (Tg) and double-Tg mouse lungs after SARS-CoV-2 infection. Representative images of the hematoxylin and eosin-stained lung sections at 0 (mock), 3, and 6 dpi. Results represent six mice per group. Images show low (left; bars, 100 μm) and high magnification (right; bars, 100 μm).

To evaluate the inflammatory responses to SARS-CoV-2 infection in the double-Tg mice, we examined the protein levels of various inflammatory cytokines and chemokines in the lungs at 0 (mock), 3, and 6 dpi using multiplex immune analysis (Fig. 4a). Inflammatory cytokine and chemokine levels were elevated mostly at 6 dpi, including those of granulocyte colony-stimulating factor (G-CSF), interferon gamma-inducible protein-10 (IP-10), MKC, monocyte chemoattractant protein-1 (MCP-1), macrophage inflammatory protein-1 (MIP-1), and interleukin-6 (IL-6), as shown in the heatmap (Fig. 4b). We also conducted histological assays to examine whether these mice had pulmonary injuries caused by SARS-CoV-2 infection. Non- and double-Tg mice were infected intranasally under the same conditions. Histopathological changes, such as alveolar wall thickening and infiltration of inflammatory cells in the lungs of double-Tg mice, were observed at 3 and 6 dpi compared with those of the non-Tg mice (Fig. 5). These findings indicated that SARS-CoV-2 infection in double-Tg mice could induce inflammatory responses in the lungs and cause acute lung injury with inflammatory cell infiltration.

### Nafamostat inhibits SARS-CoV-2 infection in double-transgenic mice

**Fig. 6:**
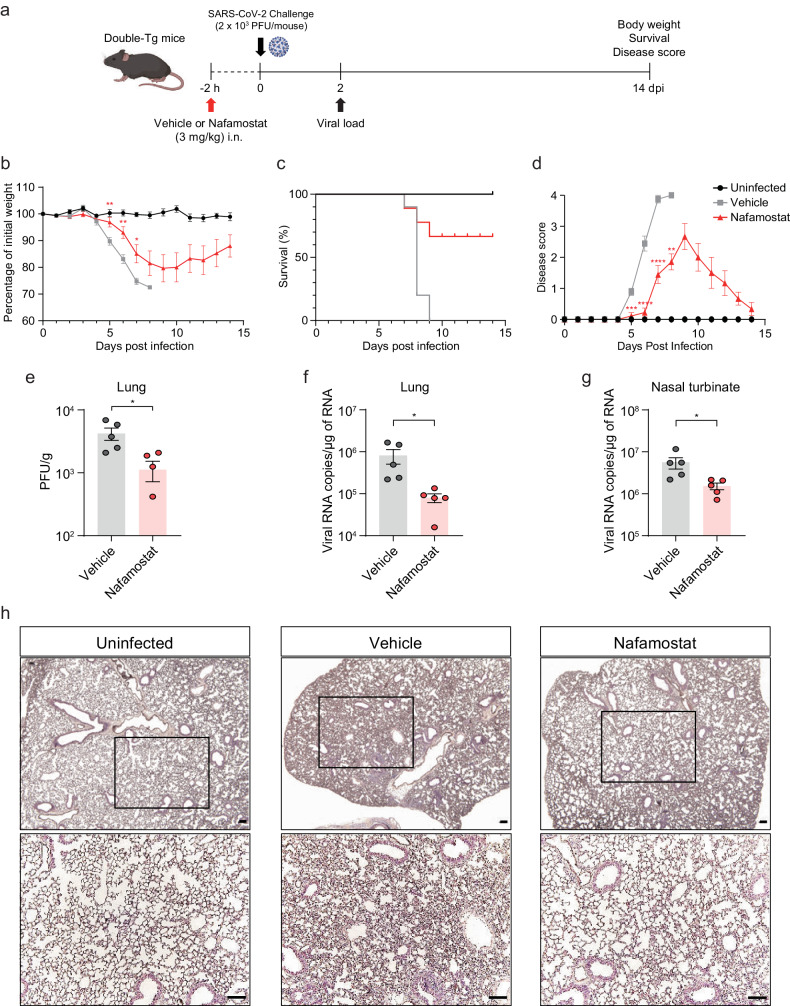
Intranasal pretreatment with nafamostat protects mice from SARS-CoV-2 infection. **a**–**h** Two hours before SARS-CoV-2 infection, mice were intranasally treated with 3 mg/kg nafamostat or 0.9% normal saline (vehicle). Weight loss (**b**), survival (**c**), and clinical disease symptoms (**d**) were monitored daily, and data were tested for significant differences using multiple *t*-tests. Infectious viral titers in the lungs (**e**) and viral loads in the lungs (**f**) and nasal turbinate (**g**) of the infected mice were detected by using a plaque assay and RT-qPCR, respectively. Symbols represent means ± SEM. **h** Representative images (*n* = 4 per group) of the hematoxylin and eosin-stained lung sections at 6 dpi. Images show low (upper; bars, 100 μm) and high magnification (lower; bars, 100 μm).

To evaluate the potential of double-Tg mice to screen and develop therapeutic agents against COVID-19, we tested the TMPRSS2 inhibitor, nafamostat, whose inhibitory activity against SARS-CoV-2 infection is lost in the absence of TMPRSS2^17^. Mice were intranasally administered 3 mg/kg nafamostat 2 h before viral infection; their body weights, survival rates, and clinical symptoms were monitored at 14 dpi, as illustrated in Fig. 6a. Nafamostat-pretreated mice lost less weight and almost recovered it by 10 dpi compared with that of vehicle-pretreated mice (Fig. 6b). Most mice survived and showed attenuated disease scores (Fig. 6c, d). These data indicated that nafamostat pretreatment effectively reduced morbidity and mortality in SARS-CoV-2-infected double-Tg mice. Owing to pretreatment, we assessed the viral load in the lungs early during infection. The infectious virus titer and viral RNA levels in the lungs and nasal turbinate were significantly reduced after nafamostat administration (Fig. 6e–g). Histopathological analysis of the lungs of infected mice at 6 dpi also revealed that nafamostat relieved the severity of lung disease caused by SARS-CoV-2 infection (Fig. 6h). These results imply that double-Tg mice could be used to develop potential therapeutic agents against COVID-19.

### Comparison of SARS-CoV-2 variant infection in the double-transgenic mice

**Fig. 7:**
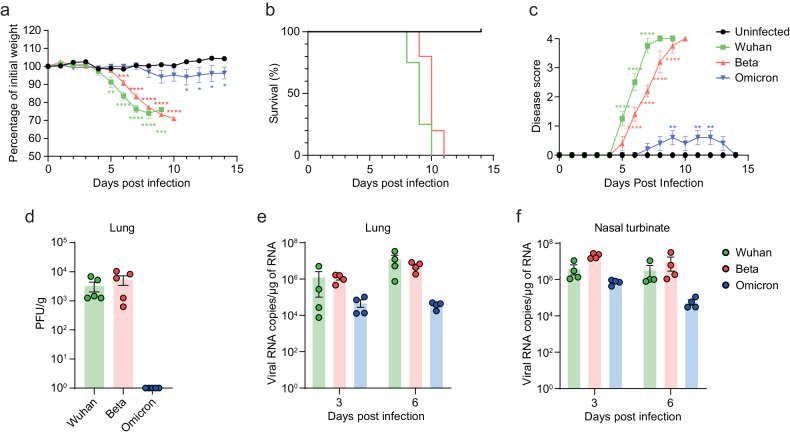
Infection of SARS-CoV-2 variants in the double-transgenic mice. **a**–**f** Mice were infected with SARS-CoV-2 Wuhan, Beta, or Omicron variant, whereafter their weight loss (**a**), survival (**b**), and clinical disease symptoms (**c**) were recorded. Statistically significant differences between the groups were determined using multiple *t*-tests. Infectious viral titers in the lungs (**d**) and viral loads in the lungs (**e**) and nasal turbinate (**f**) of the infected mice (6 dpi) were detected by using a plaque assay and RT-qPCR, respectively. Symbols represent means ± SEM.

Since its emergence in late December 2019, SARS-CoV-2 has rapidly evolved and continuously mutated, resulting in the emergence of variants with varying degrees of infectivity and lethality^18,19^. Therefore, we investigated whether double-Tg mice could demonstrate viral replication, infectivity, and pathogenicity of SARS-CoV-2 variants. To address this, we used Beta (B.1.351) and Omicron (B.1.1.529) variants, which are known to show differences in pathogenicity^20^. Infection with the Beta variant was expected to have a similar pathogenic pattern to that of the Wuhan strain, but not the Omicron variant. Double-Tg mice infected with the Beta variant showed weight loss and disease severity similar to those infected with the Wuhan strain, whereas infection with the Omicron variant did not cause morbidity and mortality (Fig. 7a–c). In agreement with other results that the Omicron variant barely replicated in lung cells^14^, we were not able to detect infectious viral particles in the lungs of the Omicron-infected mice at 6 dpi (Fig. 7d–f). These data suggested that differential pathogenesis of SARS-CoV-2 variants was demonstrated in the double-Tg mice.

## Discussion

Mice are the most commonly used experimental animals in laboratory research owing to their low cost, ease of handling, and high accessibility^2^. However, conventional wild-type mice are not susceptible to SARS-CoV-2 infection because murine ACE2 does not effectively bind to the viral spike protein^3^. Several transgenic mice expressing hACE2 have been developed as SARS-CoV-2 animal models^3,21^, followed by numerous analytical reports of immunological profiles and pathological phenotypes^22,23^. These animal models suggest that high susceptibility to SARS-CoV-2 infection is important for the evaluation of vaccines and therapeutic agents against COVID-19. Given that TMPRSS2 is involved in viral entry and supports viral infectivity (Fig. 1)^10,15^, we generated double-Tg mice expressing both hACE2 and hTMPRSS2 to increase their susceptibility to SARS-CoV-2 infection (Fig. 2). The double-Tg mice showed significant body weight loss, clinical disease symptoms, induced immune responses, and lethality in response to viral infection, indicating that they are highly susceptible to SARS-CoV-2 and could be used to develop therapeutic agents against COVID-19 (Figs. 3–5). Moreover, the antiviral effect of the TMPRSS2 inhibitor, nafamostat (Fig. 6), and vulnerability to SARS-CoV-2 variant infections were demonstrated in this model.

SARS-CoV-2 has a polybasic furin cleavage site at the S1/S2 junction of its spike protein that contributes to viral entry, infectivity, and pathogenesis. As TMPRSS2 mediates S2′ cleavage, the furin cleavage site is involved in TMPRSS2-dependent viral entry and cell tropism^9,24–26^. Absence of the furin cleavage site results in reduced viral replication and pathogenesis in animal models^16^. These studies indicated the need for hTMPRSS2 expression in a mouse model to evaluate therapeutic agents targeting viral infectivity and pathogenesis specifically. Another study showed that TMPRSS2 is essential for murine airways and plays a role in fusogenicity^14,27^. Omicron variants use TMPRSS2 inefficiently during their entry process compared with that of other variants^28^. Our study showed the attenuated pathogenesis of the Omicron variant among the other variants (Fig. 7), suggesting that double-Tg mice could be a suitable animal model for understanding viral infectivity and pathogenesis of SARS-CoV-2, especially for Omicron infections, as well as for estimating antiviral activity against COVID-19.

In summary, we confirmed that TMPRSS2 increases viral infectivity during both in vitro and in vivo transfection. We generated double-Tg mice expressing both hACE2 and hTMPRSS2 that were highly susceptible to viral infection. Our results demonstrated the pathogenicity and lethality of SARS-CoV-2 in double-Tg mice with clinical symptoms, including acute lung injury with inflammatory cell infiltration and death, recapitulating symptoms and pathology in patients with COVID-19. These findings render double-Tg mice as a useful model for understanding viral infectivity and pathogenesis and assessing potential interventions.

## Supplementary information

Supplementary Information
