# Author Correction: Generation of a lethal mouse model expressing human ACE2 and TMPRSS2 for SARS-CoV-2 infection and pathogenesis

**DOI:** 10.1038/s12276-024-01305-z

**Published:** 2024-09-04

**Authors:** Gi Uk Jeong, Insu Hwang, Wooseong Lee, Ji Hyun Choi, Gun Young Yoon, Hae Soo Kim, Jeong-Sun Yang, Kyung-Chang Kim, Joo-Yeon Lee, Seong-Jun Kim, Young-Chan Kwon, Kyun-Do Kim

**Affiliations:** 1 Center for Infectious Disease Vaccine and Diagnosis Innovation (CEVI), Korea Research Institute of Chemical Technology, Daejeon, Republic of Korea; 2 Center for Emerging Virus Research, National Institute of Health, Korea Disease Control and Prevention Agency, Cheongju, Republic of Korea; 3 Medical Chemistry and Pharmacology, University of Science and Technology (UST), Daejeon, Republic of Korea; 4 Present Address: Division of Infectious Diseases, Department of Pediatrics, Boston Children’s Hospital, Boston, MA USA; 5 Present Address: Department of Pediatrics, Harvard Medical School, Boston, MA USA; 6 Present Address: Division of Vaccine Development Coordination, Center for Vaccine Research, National Institute of Infectious Diseases, National Institute of Health, Korea Disease Control and Prevention Agency, Cheongju, Republic of Korea

**Keywords:** Viral infection, Transgenic organisms

Correction to: *Experimental & Molecular Medicine* 10.1038/s12276-024-01197-z, published online 31 May 2024

After the online publication of this article, the authors noticed an error in the Acknowledgements section. The correct statement of this article should have read as below.

“This work was supported by the Korea National Institute of Health grant (2020-ER5306 and 6634-325-210), the National Research Foundation of Korea grant (NRF-2022M3E5F1081317), and the Korea Research Institute of Chemical Technology grant (KK2333-20 and K-GRC GO! KRICT project BSF24-113).”

The authors apologize for any inconvenience caused.

The original article has been corrected.

