# Supplementary material for: Generation of a lethal mouse model expressing human ACE2 and TMPRSS2 for SARS-CoV-2 infection and pathogenesis
Source: Exp Mol Med. 2024 May 31;56(5):1221–9. doi: 10.1038/s12276-024-01197-z (PMC11148094; doi:10.1038/s12276-024-01197-z)
Supplement: Supplementary file 1 — Supplementary Information [file 12276_2024_1197_MOESM1_ESM.pdf]

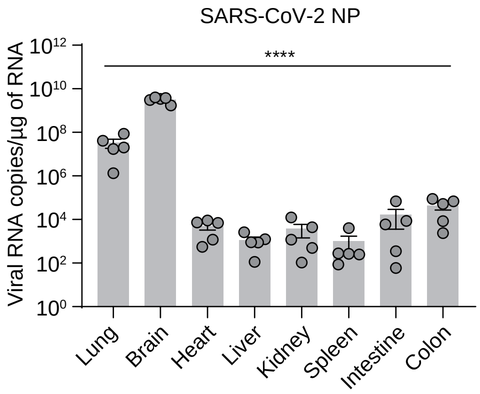

Supplementary Fig. 1 Tissue distribution of SARS-CoV-2 in the double-transgenic mice. Viral loads in the lungs, brain, heart, liver, kidney, spleen, intestine, and colon of the infected mice (6 dpi) were detected by using RT-qPCR. Symbols represent means ± SEM. Statistically significant differences between the groups were determined using one-way ANOVA.
